# Navigating the qualitative manuscript writing process: some tips for authors and reviewers

**DOI:** 10.1186/s12909-020-02370-4

**Published:** 2020-11-16

**Authors:** Chris Roberts, Koshila Kumar, Gabrielle Finn

**Affiliations:** 1 Education Office, Sydney Medical School, Faculty of Medicine and Health, The University of Sydney, Sydney, NSW Australia; 2 Prideaux Centre for Research in Health Professions Education, College of Medicine and Public Health, Flinders University, Adelaide, South Australia Australia; 3 Division of Medical Education, School of Medical Sciences, Faculty of Biology, Medicine and Health, The University of Manchester, M13 9NT, Manchester, UK

Qualitative research explores the ‘black box’ of how phenomena are constituted. Such research can provide rich and diverse insights about social practices and individual experiences across the continuum of undergraduate, postgraduate and continuing education, sectors and contexts. Qualitative research can yield unique data that can complement the numbers generated in quantitative research, [1] by answering “how” and “why” research questions. As you will notice in this paper, qualitative research is underpinned by specific philosophical assumptions, quality criteria and has a lexicon or a language specific to it.

A simple search of BMC Medical Education suggests that there are over 800 papers that employ qualitative methods either on their own or as part of a mixed methods study to evaluate various phenomena. This represents a considerable investment in time and effort for both researchers and reviewers. This paper is aimed at maximising this investment by helping early career researchers (ECRs) and reviewers new to the qualitative research field become familiar with quality criteria in qualitative research and how these can be applied in the qualitative manuscript writing process. Fortunately, there are numerous guidelines for both authors and for reviewers of qualitative research, including practical “how to” checklists [2, 3]. These checklists can be valuable tools to confirm the essential elements of a qualitative study for early career researchers (ECRs). Our advice in this article is not intended to replace such “how to” guidance. Rather, the suggestions we make are intended to help ECRs increase their likelihood of getting published and reviewers to make informed decisions about the quality of qualitative research being submitted for publication in BMC Medical Education. Our advice is themed around long-established criteria for the quality of qualitative research developed by Lincoln and Guba [4]. (see Table 1) Each quality criterion outlined in Table 1 is further expanded in Table 2 in the form of several practical steps pertinent to the process of writing up qualitative research.

**Table 1:** Five key criteria for the quality of qualitative research (adapted from Lincoln and Guba, 1995)

**Plausibility** - relates to how congruent the findings are with reality and how believable and trustworthy the research is (i.e. is the research plausible?) **Relevancy** – relates to whether others can easily determine if the findings can be applied to other settings (i.e. is the research relevant to other situations and contexts?) **Consistency** – relates to whether the study methods and procedures have been documented in a way that they can be adequately scrutinised and replicated and there is coherence between different parts of the research (i.e. is the process consistent and aligned?) **Transparency** - relates to whether the researchers have been open, explicit and clear about the methods and procedures, including changes to planned methods, and their own biases and preconceptions (i.e. is the process visible?) **Currency –** although not solely applicable to qualitative research, this relates to whether the research is appropriately situated in contemporary debate and discussion (i.e. is the research timely?)

**Table 2:** Practical steps in preparing qualitative research manuscripts

**Plausibility** • Problematise the topic by engaging with the existing literature and asking critical questions about what is not known about the phenomenon, process, or concept being studied • Articulate the significance by ensuring a research question is clearly stated and is aligned to a theoretical or empirical gap in the literature • Communicate clearly how a study has been informed by multiple perspectives (e.g. participants, methods, data sets, researchers, and/or theories) • Ensure integrity by checking resonance with participants, and reporting any subsequent changes in data interpretation • Ensure there is a coherence and logic to all parts of the narrative being presented • Outline the contributions of the research to the empirical or theoretical literature or for practice **Relevance** • Describe the study setting and outline how it provides an appropriate context for investigating the phenomenon, process, or concept being studied • Describe the sources of data and the specific characteristics of these sources relevant to the phenomenon, process, or concept being studied • Identify implications/recommendations of the research and how the research might inform other settings or populations or future work • Communicate the research using language that is meaningful for the intended audience **Consistency** • Ensure the research question/s follows logically from the literature • Outline how the choice of methods has enabled access to the phenomenon, process, or concept being studied • Describe the theoretical lens through which the findings will be interpreted • Report how the process of engaging with the literature or gathering or analysing data may have helped to fine tune the research question and the process of inquiry • Label core findings (i.e. themes) in a way that align back to the research question and are meaningful • Ensure participant quotes are used judiciously to evidence and support the findings • Review congruence by checking alignment between all sections of a manuscript and particularly between the findings and the discussion and implication points, to avoid overstatement of findings • Review coherence of the storyline by removing unnecessary literature and side topics • Utilise the correct qualitative research lexicon **Transparency** • Provide a transparent and comprehensive description of the research process that reflects key decisions or adaptations made in the process • Outline if any unexpected issues were encountered in the research process and how the researcher/s managed this • Ensure the implications/recommendations are well-grounded in the data • Provide a detailed description of the data collection and analysis processes including how these were informed by multiple researchers and theory (if applicable) • Practice reflexivity by including a statement about researcher/s background, position within the research, and relationship to the research phenomenon, context or participants • Provide a balanced view by outlining the strengths and sources of uncertainty in a study so that a reader/reviewer can make an informed judgement **Currency** • Provide a compelling reason for why the research matters, and identify 2–3 take home messages that succinctly convey the value-add of a study • Communicate about the other contexts in which the research likely matters	

As a general starting point, the early career writer is advised to consult previously published qualitative papers in the journal to identify the genre (style) and relative emphasis of different components of the research paper. Patton [5] advises researchers to “FOCUS! FOCUS! FOCUS!” in deciding which components to include in the paper, highlighting the need to exclude side topics that add little to the narrative and reduce the cognitive load for readers and reviewers alike. Authors are also advised to do significant re-writing, rephrasing, re-ordering of initial drafts, to remove faulty grammar, and addresses stylistic and structural problems [6]. They should be mindful of “the golden thread,” that is their central argument that holds together the literature review, the theoretical and conceptual framework, the research questions, methodology, the analysis and organisation of the data and the conclusions. Getting a draft reviewed by someone outside of the research/writing team is one practical strategy to ensure the manuscript is well presented and relates to the plausibility element.

The introduction of a qualitative paper can be seen as beginning a conversation. Lingard advises that in this conversation, authors need to persuade the reader and reviewer of the strength, originality and contributions of their work [7]. In constructing a persuasive rationale, ECRs need to clearly distinguish between the qualitative research phenomenon (i.e. the broad research issue or concept under investigation) and the research context (i.e. the local setting or situation) [5]. The introduction section needs to culminate in a qualitative research question/s. It is important that ECRs are aware that qualitative research questions need to be fine-tuned from their original state to reflect gaps in the literature review, the researcher/s’ philosophical stance, the theory used, or unexpected findings [8]. This links to the elements of plausibility and consistency outlined in Table 1.

Also, in the introduction of a qualitative paper, ECRs need to explain the multiple “lenses” through which they have considered complex social phenomena; including the underpinning research paradigm and theory. A research paradigm reveals the researcher/s’ values and assumptions about research and relates to axiology (what do you value?), ontology (what is out there to know?) epistemology (what and how can you know it?), and methodology (how do you go about acquiring that knowledge?) [9] ECRs are advised to explicitly state their research paradigm and its underpinning assumptions. For example, Ommering et al., state “We established our research within an interpretivist paradigm, emphasizing the subjective nature in understanding human experiences and creation of reality.” [10] Theory refers to a set of concepts or a conceptual framework that helps the writer to move beyond description to ‘explaining, predicting, or prescribing responses, events, situations, conditions, or relationships.’ [11] Theory can provide comprehensive understandings at multiple levels, including: the macro or grand level of how societies work, the mid-range level of how organisations operate; and the micro level of how people interact [12]. Qualitative studies can involve theory application or theory development [5]. ECRs are advised to briefly summarise their theoretical lens and identify what it means to consider the research phenomenon, process, or concept being studied with that specific lens. For example, Kumar and Greenhill explain how the lens of workplace affordances enabled their paper to draw “attention to the contextual, personal and interactional factors that impact on how clinical educators integrate their educational knowledge and skills into the practice setting, and undertake their educational role.” [13] Ensuring that the elements of theory and research paradigm are explicit and aligned, enhances plausibility, consistency and transparency of qualitative research. The use of theory can also add to the currency of research by enabling a new lens to be cast on a research phenomenon, process, or concept and reveal something previously unknown or surprising.

Moving to the methods, methodology is a general approach to studying a research topic and establishes how one will go about studying any phenomenon. In contrast, methods are specific research techniques and in qualitative research, data collection methods might include observation or interviewing, or photo elicitation methods, while data analysis methods may include content analysis, narrative analysis, or discourse analysis to mention a few [8]. ECRs will need to ensure the philosophical assumptions, methodology and methods follow from the introduction of a manuscript and the research question/s, [3] and this enhances the consistency and transparency elements. Moreover, triangulation or the combining of multiple observers, theories, methods, and data sources, is vital to overcome the limitation of singular methods, lone analysts, and single-perspective theories or models [8]. ECRs should report on not only what was triangulated but also how it was performed, thereby enhancing the elements of plausibility and consistency. For example, Touchie et al., describe using three researchers, three different focus groups, and representation of three different participant cohorts to ensure triangulation [14]. When it comes to the analysis of qualitative data, ECRs may claim they have used a specific methodological approach (e.g. interpretative phenomenological approach or a grounded theory approach) whereas the analytical steps are more congruent with a more generalist approach, such as thematic analysis [15]. ECRs are advised that such methodological approaches are founded on a number of philosophical considerations which need to inform the framing and conduct of a study, not just the analysis process. Alignment between the methodology and the methods informs the consistency, transparency and plausibility elements.

Comprehensively describing the research context in a way that is understandable to an international audience helps to illuminate the specific ‘laboratory’ for the research, and how the processes applied or insights generated in this ‘laboratory’ can be adapted or translated to other contexts. This addresses the relevancy element. To further enhance plausibility and relevance, ECRs should situate their work clearly on the evaluation–research continuum. Although not a strictly qualitative research consideration, evaluation focuses mostly on understanding how specific local practices may have resulted in specific outcomes for learners. While evaluation is vital for quality assurance and improvement, research has a broader and strategic focus and rates more highly against the currency and relevancy criteria. ECRs are more likely to undertake evaluation studies aimed at demonstrating the impact and outcomes of an educational intervention in their local setting, consistent with level one of Kirkpatrick’s criteria [16]. For example, Palmer and colleagues explain that they aimed to “develop and evaluate a continuing medical education (CME) course aimed at improving healthcare provider knowledge” [17]. To be competitive for publication, evaluation studies need to (measure and) report on at least level two and above of Kirkpatrick’s criteria. Learning how to problematise and frame the investigation of a problem arising from practice as research, provides ECRs with an opportunity to adopt a more critical and scholarly stance.

Also, in the methods, ECRs may provide detail about the study context and participants but little in the way of personal reflexive statements. Unlike quantitative research which claims that knowledge is objective and seeks to remove subjective influences, qualitative research recognises that subjectivity is inherent and that the researcher is directly involved in interpreting and constructing meanings [8]. For example, Bindels and colleagues provide a clear and concise description about their own backgrounds making their ‘lens’ explicit and enabling the reader to understand the multiple perspectives that have informed their research process [18]. Therefore, a clear description of the researcher/s position and relationship to the research phenomenon, context and participants, is vital for transparency, relevance and plausibility. We three are all experienced qualitative researchers, writers, reviewers and are associate editors for BMC Medical Education. We are situated in this research landscape as consumers, architects, and arbiters and we engage in these roles in collaboration with others. This provides a useful vantage point from which to provide commentary on key elements which can cause frustration for would-be authors and reviewers of qualitative research papers [19].

In the discussion of a qualitative paper, ECRs are encouraged to make detailed comments about the contributions of their research and whether these reinforce, extend, or challenge existing understandings based on an analysis that is theoretically or socially significant [20]. As an example, Barratt et al., found important data to inform the training of medical interns in the use of personal protective equipment during the COVID 19 pandemic [21]. ECRs are also expected to address the “so what” question which relates to the the consequence of findings for policy, practice and theory. Authors will need to explicitly outline the practical, theoretical or methodological implications of the study findings in a way that is actionable, thereby enhancing relevance and plausibility. For example, Burgess et al., presented their discussion according to four themes and outlined associated implications for individuals and institutions [22]. A balanced view of the research can be presented by ensuring there is congruence between the data and the claims made and searching the data and/or literature for evidence that disconfirms the findings. ECRs will also need to put forward the sources of uncertainty (rather than limitations) in their research and argue what these may mean for the interpretations made and how the contributions to knowledge could be adopted by others in different contexts [23]. This links to the plausibility and transparency elements.

## In conclusion

Qualitative research is underpinned by specific philosophical assumptions, quality criteria and a lexicon, which ECRs and reviewers need to be mindful of as they navigate the qualitative manuscript writing and reviewing processes. We hope that the guidance provided here is helpful for ECRs in preparing submissions and for reviewers in making informed decisions and providing quality feedback.
